# Serum Pentosidine in Relation to Obesity in Patients with Type 2 Diabetes and Healthy Controls

**DOI:** 10.1007/s00223-024-01338-6

**Published:** 2025-01-07

**Authors:** Sandra Baumann, Lilian Sewing, Cyril Traechslin, Wilma Verhagen-Kamerbeek, Leticia Grize, Marius Kraenzlin, Christian Meier

**Affiliations:** 1 Division of Endocrinology and Diabetes, Spital Emmental, Burgdorf, Switzerland; 2 Division of Endocrinology, Diabetes and Metabolism, University Hospital Basel, Aeschenvorstadt 57, 4051 Basel, Switzerland; 3 Swiss Tropical and Public Health Institute and University of Basel, Basel, Switzerland; 4 Endocrine Clinic and Laboratory, Basel, Switzerland

**Keywords:** Type 2 diabetes, Pentosidine, Bone turnover, Obesity

## Abstract

Pentosidine (PEN), a surrogate marker of advanced glycation end-product formation, reflects increased non-enzymatic cross-linking in bone collagen, which is thought to be an important determinant of bone fragility in type 2 diabetes mellitus (T2DM). We aimed to investigate serum concentrations of PEN in patients with T2DM and controls without T2DM and to examine its relationship with bone parameters and metabolic state such as glycaemic control, insulin resistance and body weight. In a cross-sectional study-design, data from postmenopausal women and men with T2DM (*n* = 110) and controls without T2DM (*n* = 111) were evaluated. Serum PEN was measured using an ELISA-based assay (CSB-E09415h, Cusabio). In addition, biochemical markers of glucose metabolism and bone turnover markers were measured. Bone mineral density (BMD) was assessed by dual-energy X-ray absorptiometry. After adjustment for age, gender and body mass index (BMI), serum PEN was significantly higher in patients with T2DM compared to controls (*p* = 0.02) and most prominently in women with T2DM (*p* = 0.09). We found a strong association of serum PEN concentrations with BMI in the entire study population (*R* = 0.43, *p* < 0.001) as well as in patients with T2DM (*R* = 0.28, *p* < 0.01). While bone turnover markers were significantly decreased, and BMD increased in patients with T2DM, only weak or no associations were observed between these skeletal surrogate markers and serum PEN. We conclude that serum PEN is strongly associated with BMI with highest levels in obese women with T2DM. Adjustment for patient’s weight is needed when evaluating serum PEN levels in patients with T2DM.

**Clinical Trial Information:** NCT02551315.

## Introduction

Fracture risk at trabecular and cortical bone sites is increased in patients with type 2 diabetes mellitus (T2DM), particularly in men [1]. Fracture risk assessment in T2DM remains a challenge as areal bone mineral density (BMD) is usually normal or elevated and therefore fracture risk assessment tools may underestimate fracture risk in T2DM. Furthermore, there is insufficient evidence of associations between aBMD, bone turnover markers and other bone quality parameters to identify individuals with diabetes who are at risk of non-traumatic fragility fractures [2–5]. There is an unmet need for early detection of poor bone quality and associated fracture risk, particularly in high-risk populations such as patients with T2DM.

The exact mechanisms of bone fragility in T2DM remain to be determined [6–8]. Hyperglycaemia-induced glycosylation of bone proteins may impair bone quality in patients with diabetes [9, 10]. Hyperglycaemia results in glycosylation of various proteins, including type I collagen, which has a disadvantageous effect on bone metabolism [11, 12]. The material properties of bone are regulated in part by the tissue turnover rate and the degree of oxidation and glycation. Collagen cross-linking plays an important role in the expression of bone strength. In contrast to beneficial enzymatic (lysyl oxidase regulated) cross-linking, high glucose concentrations lead to the accumulation of advanced glycation end-products (AGEs) through excessive formation of detrimental non-enzymatic cross-links [13]. Several studies have shown that there is an increase in AGEs in bone tissue from animal models of T2DM [12].

Pentosidine (PEN) is a well-established fluorescent intermolecular advanced glycation end-product (AGE) that forms crosslinks in bone collagen. Although PEN is only one of many AGEs in bone, the measurement of PEN in bone is a common method of AGE quantification. PEN can be easily and accurately measured by high performance liquid chromatography (HPLC) in small specimens, and also in serum and urine by HPLC assays or more recently by ELISA [14]. Several in vitro studies have shown that an increase in the bone content of PEN is associated with increased brittleness and decreased bone strength [15]. Serum and urine concentrations of PEN, which have been found to be higher in patients with T2DM compared to age-matched controls without T2DM [16, 17] are associated with prevalent and incident fractures either in patients with T2DM [9, 18, 19] or in postmenopausal women [9, 18, 19] and have been shown to improve risk classification using risk assessment tools such as FRAX in Japanese women. In T2DM, changes in the composition of bone material are associated with the presence of AGEs and increased bone fragility [3, 20].

Both, Vaculik et al. [21] and Odetti et al. [22] previously found a significant positive correlation between PEN measured in femoral bone samples and serum PEN concentrations. These findings suggest that measuring the levels of serum pentosidine could be used as a convenient method to mirror alterations in bone structure in patients with T2DM.

In our study, we aimed to investigate serum concentrations of PEN in a cohort of patients with T2DM and controls without T2DM and to examine its association with bone parameters and metabolic status such as glycaemic control, insulin resistance and body weight.

## Methods

### Study Population and Design

Data were obtained from the DiabOS study (NCT02551315), a multicentre, prospective, observational cohort study in patients with T2DM and controls without diabetes. The study included postmenopausal women and men (aged 50–75 years, body mass index [BMI] 18–37 kg/m^2^) who were recruited from three centres (University Hospital Basel, Kantonsspital Luzern and Kantonsspital Baselland) and through press advertisements in Switzerland between 2017 and 2020. Patients with T2DM were eligible if they had been treated with oral antidiabetics and/or insulin for at least 3 years. The study was conducted in accordance with the ethical standards of the institutional and/or national research committee and with the 1964 Helsinki declaration and its later amendments or comparable ethical standards. Informed Consent NHANES is conducted by the Centers for Disease Control and Prevention (CDC) and the National Center for Health Statistics (NCHS). The NCHS Research Ethics Review Committee reviewed and approved the NHANES study protocol. All participants signed written informed consent.

Exclusion criteria were a history of idiopathic or premenopausal osteoporosis, previous treatment with osteoporosis medication or medical conditions known to affect bone metabolism (e.g. metabolic bone disease such as primary hyperparathyroidism or Paget´s disease, metastatic bone disease, thyrotoxicosis, hypercortisolism). We also excluded persons on drugs known to have a negative effect on bone metabolism (e.g. steroids, thiazolidinediones) within 6 months prior to enrolment. Intake of oral antidiabetics other than thiazolidinediones or insulin treatment was not an exclusion criterion [23].

Study procedures included collection of clinical data on history of T2DM (time since diagnosis, diabetic complications, antidiabetic medication, insulin use), measurements of height (cm) and weight (kg) and calculation of BMI (kg/m^2^) and biochemical assessment of serum concentrations of markers of glucose metabolism (fasting plasma glucose, glycated haemoglobin [HbA1c], insulin), estimated glomerular filtration rate (eGFR), biochemical markers of bone turnover and serum concentrations of PEN. FRAX score was calculated using the online fracture risk assessment tool for Switzerland provided by the centre for Metabolic Bone Diseases at the University of Sheffield, UK, adjusted for trabecular bone score. No specific adjustments were made to account for diabetes (i.e. secondary osteoporosis). Areal bone mineral density (BMD, g/cm^2^) was assessed by dual-energy X-ray absorptiometry (DXA), and trabecular bone score (TBS) was calculated from DXA scans of the lumbar spine.

### Biochemical Assessments

Venous blood samples were taken in the morning between 08.00 and 10.00 am after an overnight fast. Serum samples were stored at − 80 ⁰C until analysis [23].

Serum HbA1c was quantified by the Afinion HbA1c assay (Abbott). Fasting glucose and insulin were measured by the automated Elecsys Insulin, cobas® (Roche Diagnostics, Germany). The HOMA-Index was calculated (HOMA-Index = fasting insulin [uU/ml] *fasting glucose [mmol/l]/22.5). Glomerular filtration rate (eGFR) was estimated using the Cockcroft-Gault equation.

Serum carboxyl-terminal cross-linking telopeptide of type I collagen (CTX) was measured by enzyme immunoassay (Elecsys^©^ β CrossLaps, Roche Diagnostics, Mannheim, Germany) with intra- and inter-assay variability of 2.0 and 8.4%, respectively. N-terminal propeptide of type I procollagen (PINP) was measured with Elecsys^©^ P1NP (Roche Diagnostics, Mannheim, Germany) with intra- and inter-assay variability of 1.2 and 3.3%, respectively. Osteocalcin (OC) was measured by the automated IDS N-MID® Osteocalcin-Assay (Immunodiagnostic Systems Ltd., UK; intra- and inter-assay variability of 1.9 and 3.1%, respectively).

Serum 25-hydroxyvitamin D (25OHVitD) and intact parathyroid hormone (iPTH) were measured by electrochemiluminescence immunoassays (ECLIA) using the cobas® e411 automated analyzer (Roche Diagnostics International, Rotkreuz, Switzerland). The intra- and inter-assay variations were 2.2% and 10.7% for 25OHVitD, and 1.2 and 2.0% for iPTH, respectively.

### Serum Pentosidine Measurement

Serum PEN was measured in duplicate by a quantitative sandwich enzyme immunoassay with an antibody specific for human pentosidine using the Research Use Only Human Pentosidine ELISA Kit (CSB-E09415h, Cusabio, www.cusabio.com) according to the manufacturer’s instructions. The assay had a standard range of 31.25–2000 pmol/mL with an intra-assay precision of < 8% and an inter-assay precision of < 10%. In our laboratory the mean intra-assay coefficient of variation (CV) was 7.2% (SD 5.1%). In 15 samples the CV between the duplicate measurements of the serum PEN concentration was > 20%. All data from these patients were excluded from the cross-sectional analysis.

### Bone Mineral Density and Trabecular Bone Score

Areal BMD was assessed at the lumbar spine, femoral neck, total hip and distal radius by dual-energy x-ray absorptiometry (DXA) using a Hologic Discovery densitometer (Horizon A (S/N 200174, Bedford MA; USA). Short term precision of the densitometer was determined by performing duplicate scans in 20 subjects. The coefficients of variation were as follows: 1.1% (lumbar spine), 1.4% (femoral neck), 1.9% (trochanteric region) and 1.1% (total hip). Device quality assurance assessments and regular machine calibrations were performed and monitored according to the manufacturer´s recommendations. Each individual DXA scan was reviewed by the investigators as an additional quality control; vertebrae with foreign bodies or degenerative changes were excluded from the BMD calculation.

TBS iNsight imaging Software (version 1,8; Med-Imaps, Pessac) was used to calculate trabecular bone score (TBS) from lumbar spine DXA scans [24].

### Statistical Analysis

Descriptive statistics were used to summarize the demographic and clinical characteristics of the study population. These are reported as mean and standard deviation (SD) for continuous parameters or frequency counts and percentage (%) for categorical parameters. The chi-squared test or Fisher’s exact test was used to compare categorical parameters in patients with T2DM and controls without T2DM. Comparisons of continuous variables such as biochemical markers and bone parameters between these two groups were performed using the Mann–Whitney *U*-test.

Pearson’s correlation coefficients were calculated to examine the association of serum PEN with age, BMI, HbA1c, diabetes duration, eGFR, P1NP, CTX, osteocalcin and BMD of the lumbar spine, total hip, femoral neck and distal radius. Generalized linear regression models were used to examine the association between serum PEN or biochemical parameters and study group (T2DM/controls) adjusting for age, gender and BMI. A *p*-value less than 0.05 (two-tailed) was considered statistically significant. Statistical analysis was performed using Statistical Analysis System 9.4 software (SAS Institute, Cary NC, USA).

## Results

### Study Population and Baseline Characteristics

A total of 221 participants in the DiabOS study had a complete baseline data set and met the inclusion criteria for this cross-sectional analysis. The demographic characteristics of patients with T2DM and controls without T2DM are summarized in Table 1. The study population was evenly distributed with 110 patients with T2DM and 111 non-diabetic controls. Patients were predominantly of Caucasian ethnicity with 214 Caucasians, 5 Asians, 1 Hispanic and 1 Black. Patients with T2DM were older (*p* < 0.001), were predominantly male (70.9%) and had a significantly higher BMI compared to controls (*p* < 0.001). Mean diabetes duration was 14.5 ± 8.1 years. Glycaemia in patients with T2DM was moderately to well-controlled with a mean HbA1c of 7.5% (7.7 ± 1.2% in men and 7.1 ± 1.2% in women).

**Table 1:** Baseline characteristics of patients with type 2 diabetes (T2DM, *n* = 110) and controls without T2DM (*n* = 111)

Characteristics	All (*n* = 221)			Men (*n* = 105)			Women (*n* = 116)		
	T2DM	Control	*p*-value^a^	T2DM	Control	*p*-value^a^	T2DM	Control	*p*-value^a^
*n* (%)	110 (49.8%)	111 (50.2%)		78 (70.9%)	27 (24.3%)	< 0.001	32 (29.1%)	84 (75.7%)	< 0.001
Age (years)	63.8 ± 6.4	60.9 ± 6.2	< 0.001	63.3 ± 6.2	61.6 ± 6.5	0.24	64.9 ± 6.6	60.6 ± 6.1	< 0.01
BMI (kg/m^2^)	29.4 ± 4.1	24.8 ± 4.4	< 0.001	29.5 ± 3.8	25.7 ± 3.2	< 0.001	29.1 ± 4.8	24.5 ± 4.7	< 0.001
Obesity (BMI ≥ 30 kg/m^2^),* n* (%)	52 (47.3%)	17 (15.3%)	< 0.001	36 (46.2%)	2 (7.4%)	< 0.001	16 (50%)	15 (17.9%)	< 0.001
HbA1c (%) Fasting glucose (mmol/L) Fasting insulin (µU/L) HOMA-Index Diabetes duration (years) Insulin therapy,* n* (%)	7.5 ± 1.2 8.0 ± 2.2 15.3 ± 15.4 5.6 ± 6.1 14.5 ± 8.1 68 (61.8%)	5.5 ± 0.3 5.2 ± 0.5 8.1 ± 8.3 2.0 ± 2.5 NA NA	< 0.001 < 0.001 < 0.001 < 0.001	7.7 ± 1.2 8.2 ± 2.1 15.7 ± 14.4 6.1 ± 6.5 14.4 ± 8.3 52 (66.7%)	5.5 ± 0.4 5.5 ± 0.5 10.9 ± 15.1 2.8 ± 4.6 NA NA	< 0.001 < 0.001 0.02 < 0.001	7.1 ± 1.2 7.5 ± 2.4 14.3 ± 17.8 4.3 ± 4.7 14.7 ± 7.7 16 (50.0%)	5.5 ± 0.3 5.1 ± 0.5 7.3 ± 4.2 1.7 ± 1.1 NA NA	< 0.001 < 0.001 0.04 < 0.01
Microvascular complications,* n* (%)	59 (53.6%)	NA		49 (62.8%)	NA		10 (31.3%)	NA	

Values presented are mean ± SD or *n* (%)

^a^ 2-tailed Mann–Whitney* U*-test was used to compare continuous parameters between two groups. Chi-Squared-test was used to compare categorical parameters between groups. NA = not applicable

Patients with T2DM were treated with metformin (81% men, 92% women), sulfonylurea (9% men, 19% women), DPP4 inhibitors (25% men, 24% women), GLP1-analogues (38% men, 43% women), SGLT2-inhibitors (29% men, 16% women) and insulin (68% men, 46% women). In a minority of male patients (1%), glucose-lowering agents were stopped during treatment course and patients were treated with diet only. Fewer women with T2DM were on postmenopausal ERT compared to controls without T2DM (15.6 and 26.2%, *p* = 0.12).

### Biochemical and Bone Turnover Parameters

After adjustment for age, gender and BMI, renal function determined by eGFR was comparable in patients with T2DM and controls. There were no differences in 25OH-Vitamin D and iPTH levels between the two groups. Biochemical markers of bone formation (PINP, OC) and bone resorption (CTX) were lower in patients with T2DM compared to controls (P1NP −34.8%, CTX −41.5% and OC −42.0%, *p* < 0.001) and remained significantly lower after adjusting for age, gender and BMI (Table 2).

**Table 2:** Association between biochemical parameters, serum pentosidine, bone turnover markers and BMD and study group, adjusted by age, gender and BMI

	Control (*n* = 111)			T2DM (*n* = 110)			
Characteristics	Adjusted mean	95% CI for adjusted mean	SD	Adjusted mean	95% CI for adjusted mean	SD	*P*
eGFR (ml/min)	86.14	83.09 to 89.18	1.55	85.96	82.96 to 88.96	1.52	0.94
iPTH (ng/L)	45.45	41.94 to 48.95	1.78	39.20	35.72 to 42.69	1.77	0.03
IGF1 (ng/mL)	117.89	109.95 to 125.83	4.03	119.57	111.84 to 127.30	3.92	0.79
25OHVitD (mmol/L)	65.18	59.74 to 70.62	2.76	60.17	54.80 to 65.54	2.72	0.25
Pentosidine (pmol/mL)	721.87	677.94 to 765.79	22.29	798.51	755.28 to 841.74	21.93	0.03
P1NP (ng/mL)	54.85	50.81 to 58.89	2.05	37.95	34.01 to 41.90	2.00	< 0.0001
CTX (ng/mL)	0.40	0.37 to 0.44	0.02	0.280	0.25 to 0.31	0.02	< 0.0001
Osteocalcin (ng/mL)	18.13	16.69 to 19.56	0.73	11.57	10.28 to 12.87	0.66	< 0.0001
Lumbar spine BMD (g/cm^2^)	0.964	0.933 to 0.996	0.02	1.024	0.993 to 1.055	0.02	0.02
Femoral neck BMD (g/cm^2^)	0.762	0.738 to 0.785	0.01	0.800	0.772 to 0.820	0.01	0.07
Total hip BMD (g/cm^2^)	0.928	0.903 to 0.953	0.01	0.975	0.951 to 1.000	0.01	0.02
Distal radius BMD (g/cm^2^)	0.577	0.562 to 0.592	0.01	0.598	0.583 to 0.613	0.01	0.07
Trabecular Bone Score	1.313	1.288 to 1.338	0.01	1.286	1.262 to 1.310	0.01	0.18

### Bone Mineral Density and TBS

After adjustment for age, gender and BMI, BMD was significantly higher at the lumbar spine and total hip in patients with T2DM. At the femoral neck and distal radius, there was a trend towards higher BMD in patients with T2DM. TBS was comparable between the two groups (Table 2).

### Serum Pentosidine in T2DM and Controls

**Fig. 1:**
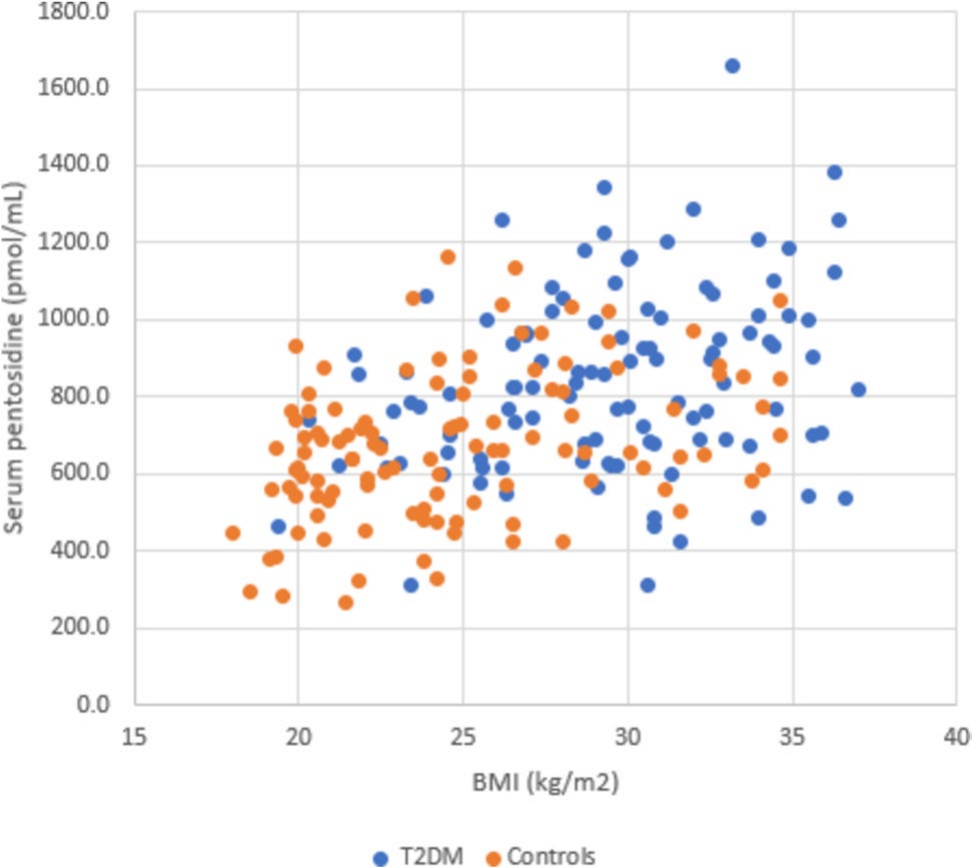
Distribution of serum pentosidine concentrations (pmol/mL) and BMI in patients with T2DM (blue dots) and non-diabetic controls (orange dots); *r* = 0.43, *p* < 0.001

In our univariate analysis of the entire study population, serum PEN concentrations were significantly associated with BMI (*R* = 0.43, *p* < 0.001, Fig. 1), HbA1c (*R* = 0.31, *p* < 0.001) and fasting glucose concentrations (R = 0.27, p < 0.001).

We also found a significant negative association of serum PEN levels with bone turnover markers P1NP (*R* =  − 0.18, *p* < 0.01), CTX (*R* =  − 0.22, *p* < 0.01) and osteocalcin (*R* =  − 0.24, *p* < 0.01) and a significant positive association with BMD at the femoral neck, hip and distal radius in the entire population (Table 3). Based on regression analysis, serum PEN concentrations were significantly higher in T2DM than in controls after adjusting for age, gender and BMI (*p* = 0.0279, Table 2). In the subgroup of patients with T2DM, PEN concentrations were only associated with BMI (*R* = 0.28, *p* < 0.01). No association was observed between serum PEN levels and disease duration or glycaemic control. Similarly, no correlation was found between PEN and bone turnover markers and BMD at different sites in T2DM. No association was found between PEN and age in the entire population or in the subgroup of T2DM.

**Table 3:** Associations between serum pentosidine concentrations and BMI, BMD and biochemical and bone turnover parameters in the entire DiabOS population (n = 221)

	Estimate	SE	Pearson’s R	*p*
BMI (kg/m^2^)	20.7	2.9	0.43	< 0.001
HbA1c (%)	55.3	11.1	0.31	< 0.001
Fasting glucose (mmol/L)	29.9	7.4	0.27	< 0.001
P1NP (ng/mL)	− 1.93	0.73	− 0.18	< 0.01
CTX (ng/mL)	− 280.4	85.1	− 0.22	< 0.01
Osteocalcin (ng/mL)	− 8.2	2.5	− 0.24	< 0.01
Lumbar spine BMD (g/cm^2^)	181.0	99.3	0.13	0.07
Femoral neck BMD (g/cm^2^)	269.0	130.1	0.14	0.04
Hip BMD (g/cm^2^)	367.8	111.3	0.22	< 0.01
Distal radius BMD (g/cm^2^)	549.5	173.6	0.21	< 0.01

**Fig. 2:**
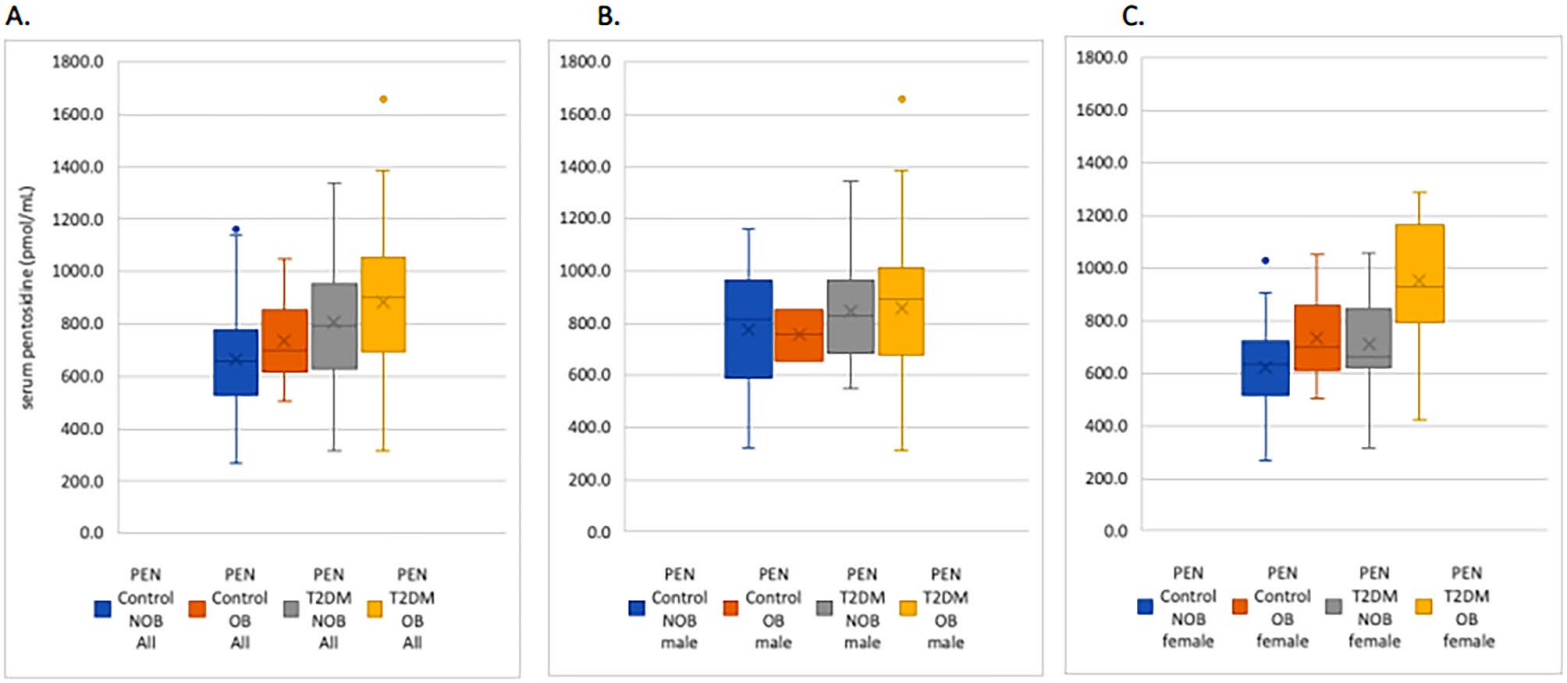
Serum pentosidine concentrations (pmol/mL) stratified by study group and by obesity: **a** the entire study cohort, **b** men and **c** women. Abbreviations: NOB, non-obese (BMI < 30 kg/m^2^); OB, obese (BMI ≥ 30–39.9 kg/m.^2^)

When we divided our patients into subgroups according to BMI (BMI > 30 kg/m^2^, BMI < 30 kg/m^2^) and gender, we found that PEN levels were highest in obese women with T2DM (Cohen’s D: 0.16, 95% CI 0.0732–0.2690 *p* = 0.09), but less so in men (Cohen’s D: 0.02, 95% CI − 0.0000 to 0.0871; *p* = 0.33) (Fig. 2).

## Discussion

Based on our cross-sectional study we confirm that serum PEN levels are increased in patients with T2DM. Importantly, serum PEN is significantly associated with BMI and to a lesser extent with glycaemic control with the highest PEN levels in obese women with T2DM. Whereas bone turnover markers were significantly decreased, and BMD increased in patients with T2DM, only weak or no associations were observed between these skeletal surrogate markers and serum PEN.

In our study we observed higher serum PEN levels in patients with T2DM compared to controls without T2DM. This is in line with previous findings [16, 17], but in contrast to Hunt et al., who could not show an effect of diabetes on serum PEN levels [4]. In our patients with T2DM, serum PEN was only associated with BMI and independent of glycaemic status (HbA1c, fasting glucose and HOMA-Index), patient age and disease duration. This may be explained by our moderately to well-controlled T2DM patients within a narrow HbA1c range. Piccoli et al. were able to show that even in well-controlled T2DM patients with HbA1c of 6.5 ± 1.7% there is a 1.5-fold increase in PEN in the bone of T2DM patients compared to controls [25]. In the whole study population, the effect of obesity on serum PEN was most pronounced in women, with obese women with T2DM having the highest serum PEN concentrations. We hypothesize that both, in relatively well-controlled patients with T2DM and in obese controls without T2DM, formation of AGEs may lead to deposition of AGEs in bone.

Our results indicate that serum PEN concentrations need to be interpreted in the context of a patient’s weight and metabolic status. Based on the significant association between serum PEN and BMI in both study groups, we suggest that there may be an additional diabetes-independent and obesity-related mechanism of PEN deposition in the bone of obese patients.

In addition to the endogenous formation of PEN via hyperglycaemia-induced, non-enzymatic glycation of macromolecules in patients with diabetes, the receptor for advanced glycation end-products (RAGE) appears to play an important role in the accumulation of PEN in obese patients. RAGE expression is increased in adipose tissue of obese patients and the activation of RAGE (i.e. as AGE-RAGE interaction) can modulate and maintain inflammatory processes via the AGE-RAGE signalling pathway. This AGE-RAGE signalling pathway promotes intracellular synthesis of reactive oxygen species (ROS), which, via post-translational modifications of proteins, increase glycoxidative products such as PEN and carboxymethyl lysine (CML) [26–28]. These pathogenetic explanations are supported by a preclinical study in mice by Stephen et al. [29]: The authors investigated the relationship between PEN accumulation in bone matrix and bone quality in obesity, focusing on the role of RAGE. Female wildtype (WT) mice on a high-fat diet showed increased levels of AGEs (PEN and CML) and reduced bone quality compared to those on a low-fat diet. In contrast, female RAGE knockout (KO) mice, maintained bone quality despite weight gain, with reduced PEN accumulation. Males showed no significant changes in bone quality. The authors concluded that high-fat diets are more detrimental to bone health in females, that the sex differences may be due to interactions between inflammation and sex hormones such as oestrogen in overweight mice, and that removing RAGE can alleviate these effects.

Apart from endogenous formation, PEN (and other AGEs) can also be ingested exogenously from the diet and then subsequently metabolized [30]. In recent years, numerous studies have investigated the AGE content of dietary sources and have shown that a modern Western diet, high in processed foods, fats and refined carbohydrates, contains elevated levels of AGEs [31, 32]. In support of this finding Foroumandi et al. found a negative correlation between serum PEN and diet quality (as assessed by the Dietary Quality Index-International) [33].

In view of the above findings, we hypothesize that the positive association of serum PEN concentrations with BMI in our study cohort as a whole and in the T2DM subgroup may be related, on the one hand, to an increased generation of PEN through an inflammatory state with persistent oxidative stress, partly due to the AGE-RAGE interaction and, on the other hand, to poor dietary habits. Furthermore, the association between PEN and obesity was strongest in women, which could be due to an interaction between inflammation and sex hormones in adipose tissue, as suggested by Stephan et al. [29]. Further studies are needed to better understand bone metabolism in obesity and its influence on PEN.

In contrast to other studies, we did not find an association of PEN with diabetes duration [25, 34] (possibly due to our well-controlled T2DM patients), nor an age-related increase in serum PEN [17, 35] in the 50–75 age group (possibly related to a relatively homogenous age structure). In accordance with the findings of Kida et al., we suggest that in our population (aged over 50 years), the greater variance in PEN levels due to additional factors (such as obesity, different lifestyle, comorbidities) may have contributed to the lack of association of PEN with age in our study [36].

In our cohort of patients with T2DM, we were not able to confirm the results of previous studies [9, 37, 38] showing a negative association of PEN with BMD and/or TBS. We did not observe any association between serum PEN and BMD at different measuring sites irrespective of the presence of T2DM and/or obesity. We attribute this to our better controlled T2DM patients (HbA1c 7.5 ± 1.2%) compared to the other studies (e.g. Schwartz et al., 8.1 ± 1.5%), possibly resulting in less hyperglycaemia-induced non-enzymatic cross-linking and thus less accumulation of PEN. However, the lack of association was not entirely unexpected and may highlight the fact that BMD is often normal in T2DM despite impaired bone quality, which is due to altered mechanical properties.

When analysing the association of serum PEN with bone turnover markers, we found a negative correlation with P1NP, CTX and OC in the whole study population. Accordingly, Furst et al. showed a negative correlation between measured skin autofluorescence (SAF) pentosidine and P1NP in postmenopausal women with T2DM [39]. Low bone turnover is thought to be a consequence of the reduced bone formation mediated by AGE-RAGE interactions and their signalling pathway, promoting variable inflammatory processes and ROS synthesis [26, 40]. Interestingly though, our study did not find an association between serum PEN and bone turnover markers in the T2DM group, which, may be due to our well-controlled T2DM patients, as previously hypothesized.

Our study has several limitations. The cross-sectional design does not allow for conclusions about a causal relationship between T2DM and/or obesity and increased serum PEN concentrations on bone characteristics. Moreover, patients with T2DM were well-controlled with a mean HbA1c of 7.5% and we found no association between serum PEN and HbA1c and disease duration.

The clinical utility of serum PEN is limited by both analytical noise on the one hand and a large biological variation in the study population of 50–75-year-old well-controlled individuals with and without T2DM. Nevertheless, the analyses in our DiabOS cohort identify trends and associations that warrant further investigation. Further studies are needed to determine the critical cut-off value of serum PEN to diagnose and monitor clinically relevant changes in bone properties in obese individuals with and without T2DM.

Due to the small number of patients with fragility fractures in the DiabOS study, the cross-sectional cohort focused on patients and controls without fragility fractures. Therefore, we could not determine whether there is an association between serum PEN and the occurrence of fragility fractures.

In conclusion we confirm that serum PEN levels are increased in patients with T2DM. Importantly, serum PEN is significantly associated with BMI and to a lesser extent with glycaemic control with the highest levels in obese women with T2DM. In addition, serum PEN was elevated in obese controls without T2DM. While bone turnover markers were significantly decreased, and BMD increased in patients with T2DM, only weak or no associations were observed between these skeletal surrogate markers and serum PEN. Based on our results adjustments for patient’s weight are needed when evaluating serum PEN levels in patients with T2DM.
