# Correction to: Serum Pentosidine in Relation to Obesity in Patients with Type 2 Diabetes and Healthy Controls

**DOI:** 10.1007/s00223-025-01352-2

**Published:** 2025-02-24

**Authors:** Sandra Baumann, Lilian Sewing, Cyril Traechslin, Wilma Verhagen-Kamerbeek, Leticia Grize, Marius Kraenzlin, Christian Meier

**Affiliations:** 1 Division of Endocrinology and Diabetes, Spital Emmental, Burgdorf, Switzerland; 2 Division of Endocrinology, Diabetes and Metabolism, University Hospital Basel, Aeschenvorstadt 57, 4051 Basel, Switzerland; 3 Swiss Tropical and Public Health Institute and University of Basel, Basel, Switzerland; 4 Endocrine Clinic and Laboratory, Basel, Switzerland

**Correction to: Calcified Tissue International** 10.1007/s00223-024-01338-6

In this article, the Fig. 2 was appeared incorrectly; the correct figure should have appeared as shown below.

The original article has been corrected.

**Incorrect Fig. 2:** 
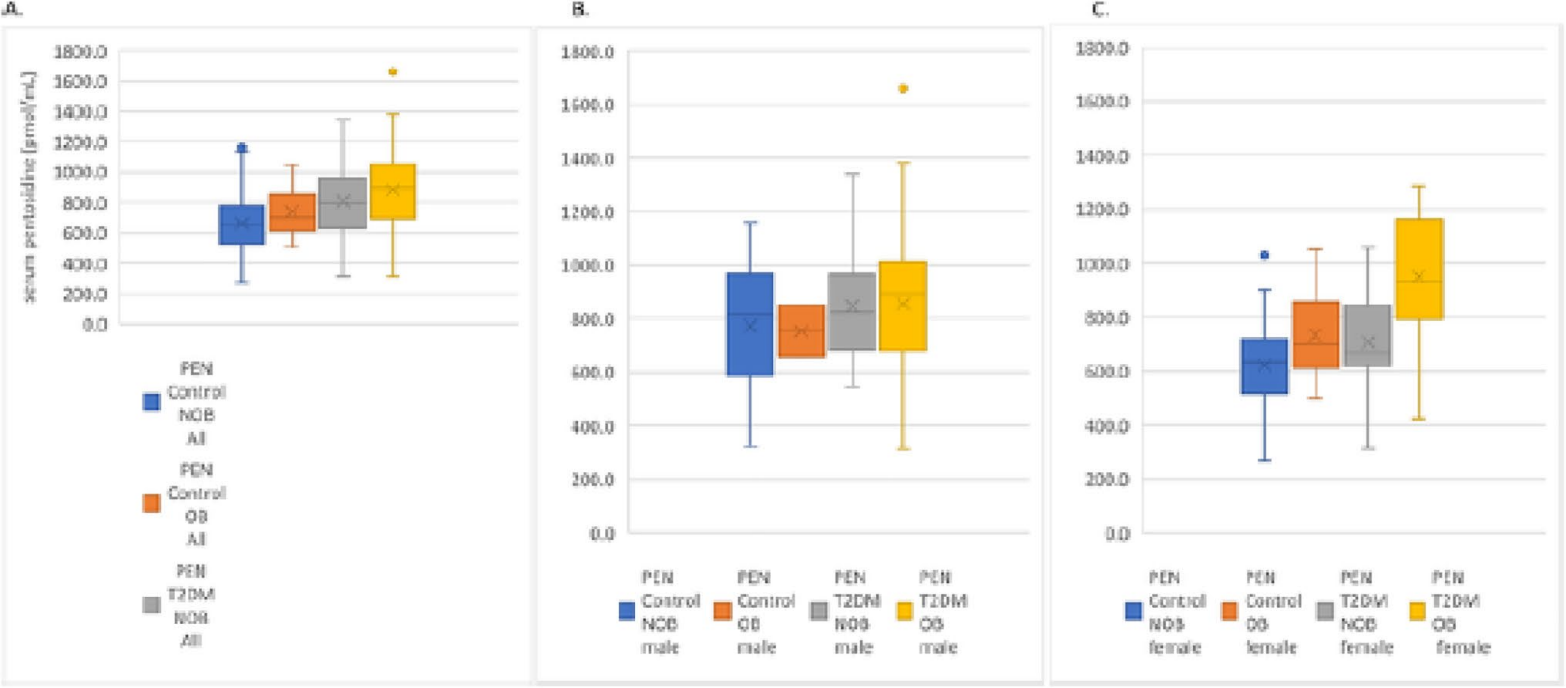


**Fig. 2** Serum pentosidine concentrations (pmol/mL) stratifed by study group and by obesity: **a** the entire study cohort, **b** men and **c** women. Abbreviations: NOB, non-obese (BMI ≥ 30–39.9 kg/m.2

**Correct Fig.** 2**:**

**Fig. 2:**
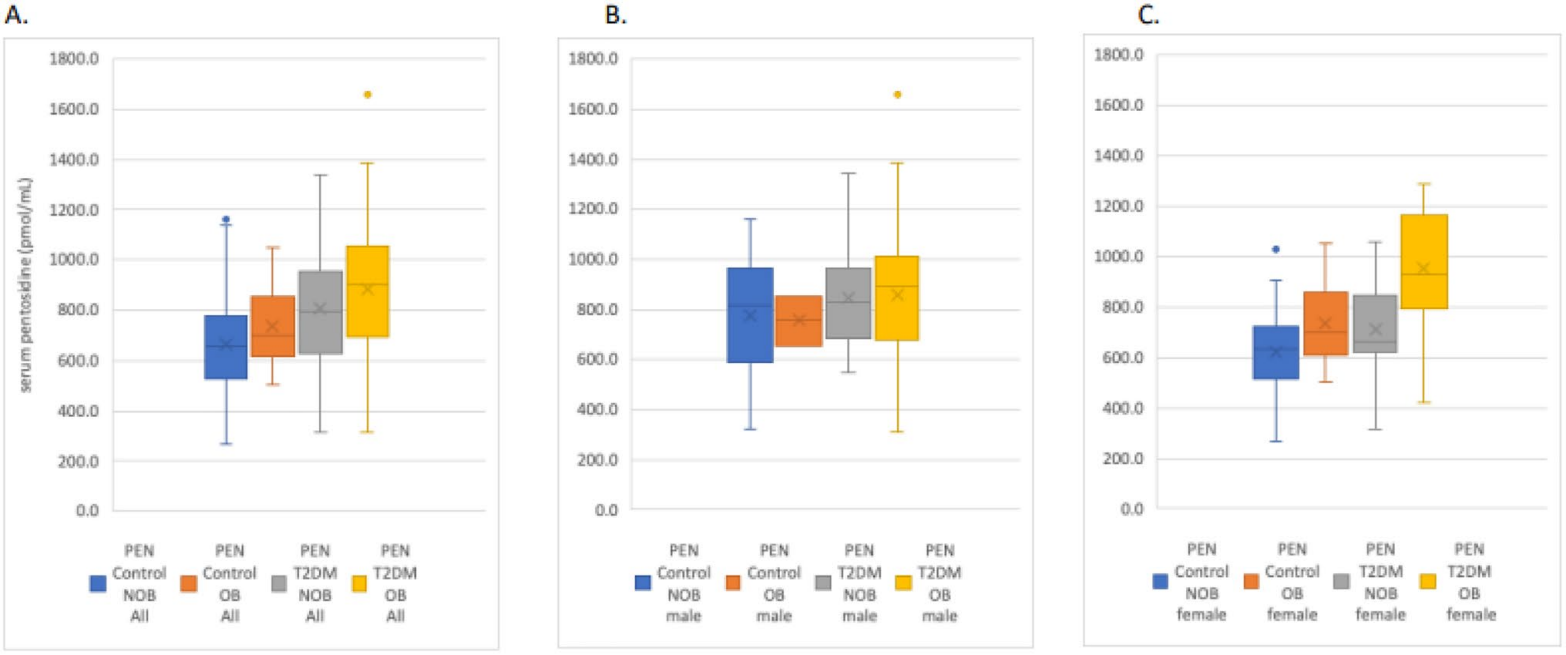
Serum pentosidine concentrations (pmol/mL) stratified by study group and by obesity: **a** the entire study cohort, **b** men and **c** women. Abbreviations: NOB, non-obese (BMI ≥ 30–39.9 kg/m^2^)

